# Nature-Positive for Industrial Facilities: An Environmental Management Framework for Biodiversity Stewardship

**DOI:** 10.1007/s00267-026-02601-2

**Published:** 2026-09-01

**Authors:** Ángela Martín-Méndez, Francisco Campuzano-Bolarín, David López-López

**Affiliations:** 1 Department of Veterinary Medicine, Faculty of Biomedical and Health Sciences, Universidad Europea de Madrid (UEM), Madrid, Spain; 2 Business Economy Department, Universidad Politécnica de Cartagena (UPCT) Member of European University of Technology EUT+, Campus Muralla del Mar, Cartagena, Spain; 3 ESADE Business School, Barcelona, Spain

**Keywords:** Biodiversity stewardship, Industrial facilities, Environmental management, Environmental management systems, Nature-positive, Mitigation hierarchy

## Abstract

Biodiversity has become an increasingly important concern in environmental management, yet the meaning of nature-positive remains unstable when applied to the management of industrial facilities. Site managers are increasingly expected to demonstrate contributions to biodiversity improvement beyond compliance, but practical guidance remains limited on what facilities can credibly implement, maintain, measure, and communicate. This article addresses this management problem through a structured critical review of the literature across six connected domains: nature-positive, no net loss and net gain approaches, the mitigation hierarchy and biodiversity offsetting, biodiversity measurement and reporting, environmental management systems (EMS), and industrial and SME implementation contexts. To improve transparency, the review is organised around an explicit, theme-led search and analytical coding process focusing on definitions, netting logic, controllability, measurement, governance mechanisms, and reporting implications. The review identifies four recurrent tensions that constrain facility-scale biodiversity stewardship: ambiguity between nature-positive as a societal goal and as a site-level claim; the methodological fragility of net-outcome claims at facility scale; limited managerial control over ecological outcomes; and weak persistence when biodiversity actions are not embedded in operational routines. Based on these findings, the paper develops Nature-Positive for Industrial Facilities (NP4IF) as a facility-scale implementation architecture for biodiversity stewardship. NP4IF specifies a PDCA-compatible sequence linking site assessment, prioritisation of controllable interventions, operational integration, two-layer monitoring, governance review, reporting discipline, and adaptive management. Its distinctive contribution is to translate nature-positive ambition into a bounded management process in which facilities are accountable for implemented and maintained actions, while ecological signals are interpreted cautiously in light of partial controllability and wider landscape dynamics. The article offers an internationally relevant, practice-oriented framework for industrial facilities and provides a more credible basis for environmental management than abstract net-outcome claims alone.

## Introduction

**Fig. 1:**
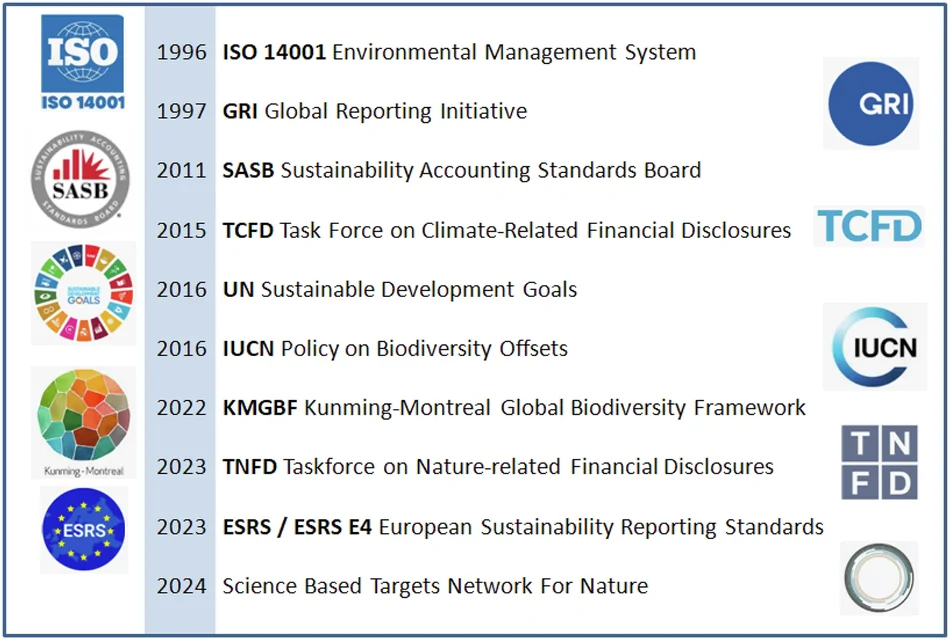
Historical evolution of environmental management and sustainability governance. Source: authors’ elaboration based on ISO 14001, GRI, SASB, TCFD, UN SDGs, IUCN, KMGBF, TNFD, ESRS E4, and SBTN documentation

Biodiversity has become a central issue in environmental governance and environmental management. The Kunming-Montreal Global Biodiversity Framework (KMGBF), the Taskforce on Nature-related Financial Disclosures (TNFD), the European Sustainability Reporting Standards (ESRS), and science-based target initiatives have increased expectations for firms to identify, manage, and communicate biodiversity-related impacts, dependencies, risks, and opportunities (Convention on Biological Diversity 2022; TNFD 2023; European Union 2023; Science Based Targets Network 2024). Within this context (Fig. 1), nature-positive has emerged as a prominent but contested concept, used as a global goal, policy direction, and corporate commitment (Locke et al. 2021; Milner-Gulland 2022; Obura et al. 2023; White et al. 2024; Buckley et al. 2025; Stenseke 2025).

For industrial facilities, the main challenge is practical. Factories, logistics platforms, energy sites and similar managed sites are increasingly expected to contribute to biodiversity improvement beyond compliance, yet managers often lack clear guidance on what can credibly be implemented, maintained, monitored, and communicated at the facility scale (Rainey et al. 2015; de Silva et al. 2019; Krause et al. 2020; Morrison-Saunders and Sánchez 2024). This matters because many relevant pressures are local and operational, including vegetation management, lighting, drainage, chemical use, disturbance, and contractor routines.

This difficulty is intensified by the close association between nature-positive and earlier net-based approaches, such as no net loss, net positive impact, and biodiversity net gain. Although these approaches appear to offer a familiar logic based on baselines, quantified losses and gains, and net results, the literature shows that biodiversity is multidimensional, place-dependent, and difficult to reduce to universally commensurable units. Questions of equivalence, additionality, timing, permanence, attribution, and governance remain unresolved in offsetting and net-gain debates (Bull et al. 2013; Maron et al. 2016; Bull and Strange 2018; Arlidge et al. 2018; Bull et al. 2020; Kujala et al. 2022; Maron et al. 2024; Wauchope et al. 2024).

At the facility scale, these methodological problems are compounded by limited controllability. Managers can influence habitat condition through actions such as altered mowing regimes, planting design, drainage management, lighting attenuation, and reduced chemical pressure, but they cannot fully control species occurrence, persistence, or long-term ecological recovery, which also depend on broader landscape and temporal dynamics (Rainey et al. 2015; Victurine et al. 2024; Pardo et al. 2025). This has direct implications for monitoring and communication, because strong outcome claims may overstate what site-level interventions can realistically deliver (Boiral 2016; Blanco-Zaitegi et al. 2022; Senanayake et al. 2024).

This article addresses the problem through a structured critical review of six connected domains: nature-positive; net-based biodiversity approaches; the mitigation hierarchy and offsetting; biodiversity measurement and reporting; environmental management systems (EMS); and industrial and SME implementation contexts. The objectives are threefold: (i) to clarify how nature-positive has been defined in relation to adjacent frameworks; (ii) to assess the main constraints on facility-scale biodiversity claims; and (iii) to derive implications for environmental management practice.

On this basis, the paper develops Nature-Positive for Industrial Facilities (NP4IF) as a facility-scale implementation architecture for biodiversity stewardship. NP4IF links site assessment, prioritisation of controllable interventions, operational integration, two-layer monitoring, governance review, reporting discipline, and adaptive management. Its contribution is to translate nature-positive ambition into a bounded management process in which facilities are accountable for implemented and maintained actions, while ecological signals are interpreted cautiously in light of partial controllability and wider landscape dynamics.

For the purposes of this article, industrial facilities are understood as bounded, managed sites where firms exercise direct or indirect control over land, infrastructure, maintenance routines, contractors, and operational pressures. This includes manufacturing plants, logistics centres, energy and utility sites, industrial parks, and SMEs with managed outdoor areas. These facilities differ in ecological potential, available space, regulatory exposure, and technical capacity, but they share a common management condition: biodiversity-related action must be compatible with safety, production, maintenance, compliance, and cost constraints. The framework is not intended as a stand-alone model for large extractive landscapes, protected-area management, landscape-scale restoration programmes, biodiversity markets, or corporate-level net accounting. These wider models may be relevant as complementary governance mechanisms, but the primary focus of NP4IF is the facility perimeter as the unit of action, evidence, responsibility, and claim (Rainey et al. 2015; de Silva et al. 2019; Morrison-Saunders and Sánchez 2024).

## Methods: Structured Critical Review

This article adopts a structured critical review design because the question it addresses is conceptual, managerial, and interdisciplinary, rather than focused on a single intervention or standardised outcome. The review examines how nature-positive can be interpreted for industrial facilities in ways that are credible for environmental management practice. A conventional meta-analysis was not appropriate because the field spans biodiversity governance, conservation policy, corporate reporting, environmental management systems (EMS), and industrial implementation, and lacks a common outcome variable, study design, or unit of analysis. The review was therefore designed to support three objectives: to clarify how nature-positive has been defined in relation to adjacent frameworks; to assess the main constraints on facility-scale biodiversity claims; and to derive implications for environmental management practice.

### Review Design and Justification

The search and source-identification process followed a structured, theme-led strategy. The literature was identified primarily through Scopus, Web of Science, and Google Scholar, complemented by targeted retrieval of key policy and standards documents. Search terms included combinations of “nature-positive”, “no net loss”, “net positive impact”, “biodiversity net gain”, “mitigation hierarchy”, “biodiversity offsets”, “biodiversity reporting”, “industrial facility”, “environmental management system”, and “SME”.

The review was organised around six connected domains: nature-positive; net-based biodiversity approaches; the mitigation hierarchy and offsetting; biodiversity measurement, accounting, and reporting; EMS and continuous-improvement literature; and industrial, infrastructure, and SME implementation contexts. Particular attention was given to literature published after the KMGBF, while foundational contributions were retained where necessary to establish the main conceptual and methodological debates (Convention on Biological Diversity 2022; Bull et al. 2013; Boiral 2007; Boiral 2016; Waxin et al. 2019; Kangas et al. 2021; Obura 2023; White et al. 2024).

Sources were included if they made a direct analytical contribution to at least one of these domains and offered implications for environmental management, implementation, governance, measurement, or biodiversity claims. The corpus therefore combined peer-reviewed literature with selected institutional and policy documents, including the KMGBF, TNFD recommendations, ESRS, science-based target guidance, and selected biodiversity offset and net gain documents, because these texts actively shape managerial expectations and reporting pressures (Convention on Biological Diversity 2022; TNFD 2023; European Union 2023; IUCN 2016; IUCN 2023; Science Based Targets Network 2024). Sources were excluded if biodiversity was only peripheral, if the argument was mainly promotional, or if highly localised observations offered little relevance beyond a specific case.

Selection proceeded on the basis of conceptual relevance and analytical filtering rather than formal quality scoring. This approach was appropriate because the evidence base includes conceptual papers, empirical studies, policy texts, and governance documents that are not directly comparable using a single appraisal tool. Priority was given to sources that defined or critiqued nature-positive, examined the integrity of netting and offsetting, addressed biodiversity metrics and reporting risks, or discussed EMS, continuous improvement, and implementation in industrial and SME settings (de Silva et al. 2019; Milner-Gulland 2022; Morrison-Saunders and Sánchez 2024; Wauchope et al. 2024; White et al. 2024; Bull et al. 2025; Stenseke 2025). Foundational works were retained where they established the core debates on offsets, no net loss, biodiversity reporting, and EMS implementation (Bull et al. 2013; Boiral 2007; Boiral 2016).

Each source was then read against a common set of analytical questions: how nature-positive was defined; whether a netting logic was used; how the mitigation hierarchy, offsetting, equivalence, additionality, timing, and permanence were treated; what assumptions were made about controllability and attribution; how measurement, monitoring, accounting, and reporting were addressed; and what management mechanisms were proposed for implementation. This coding enabled comparison across literatures that do not usually interact directly. The synthesis proceeded in two stages: first, by identifying recurrent tensions that constrain facility-scale biodiversity stewardship; and second, by deriving nature-positive for Industrial Facilities (NP4IF) as a facility-scale environmental management framework grounded in the reviewed literature rather than proposed independently of it. Source identification ended when additional searches within the six thematic domains no longer produced substantively new concepts, constraints, or implementation mechanisms relevant to facility-scale biodiversity stewardship.

### Scope and Limitations of the Review

The reviewed literature includes different positions on nature-positive, offsetting, biodiversity markets, and corporate claims. Some contributions emphasise the practical need for measurable targets, disclosure, and market-based mechanisms, whereas others adopt a more precautionary position, warning that offsetting, netting, and nature-positive claims may obscure ecological uncertainty, weak equivalence, or governance failures. This review does not treat any one position as self-evident. Instead, it uses this diversity of perspectives to derive a facility-scale framework that is operationally useful while cautious about claims that exceed managerial control (Bull et al. 2013; Maron et al. 2016; Boiral 2016; Damiens et al. 2021; Maron et al. 2024; Morrison-Saunders and Sánchez 2024; Wauchope et al. 2024; Bull et al. 2025).

This review is structured and critical rather than fully systematic, and it does not claim exhaustive coverage of all biodiversity-related publications. Its purpose is not to provide a comprehensive bibliometric census of the field, but to deliver a transparent and analytically bounded synthesis of the strands of literature most relevant to environmental management at industrial facilities. The corpus combines peer-reviewed and institutional sources. This improves practical relevance, because institutional frameworks and reporting standards materially shape managerial expectations, but it also reduces formal comparability across sources. In addition, the literature reviewed is weighted towards Anglophone and European debates, which should be recognised when considering the international transferability of specific discussions or examples. Even so, the review provides a transparent and disciplined synthesis of the literatures most directly relevant to the operational and communicative challenges of facility-scale biodiversity stewardship.

### Terminological Note

The distinction between biodiversity, habitat condition, ecological function, and ecosystem condition follows the need for greater precision in biodiversity accounting and ecosystem assessment as shown in Table 1.

**Table 1:** List of main terms and its meaning in this study

Term	Meaning in this study	Use in NP4IF
Biodiversity	Variability among living organisms, including diversity within species, between species, and of ecosystems (Convention on Biological Diversity 2022; United Nations 2021)	The broad concept that NP4IF seeks to support; it is not treated as a single directly measurable variable.
Habitat condition	State of the physical and biological features of a site that support species and ecological processes, including vegetation structure, soil, water, shelter, and disturbance levels (United Nations 2021; Nicholson et al. 2021; Marshall et al. 2024)	The main facility-scale management focus because it can be influenced more directly than species occurrence or population recovery.
Ecological function	Ecological roles and processes supported by the site, such as refuge provision, connectivity, pollination support, and reduced disturbance (Nicholson et al. 2021; Ferrier 2024; Obura et al. 2023)	Used to interpret how interventions may create ecological value beyond simple greening.
Ecosystem condition	Quality of an ecosystem in terms of composition, structure, function, and integrity (United Nations 2021; Nicholson et al. 2021; Obura et al. 2023)	Broader reference concept; NP4IF may contribute locally but does not claim full ecosystem-level recovery.
Ecosystem recovery	Process through which ecosystem condition, integrity, resilience, and natural processes improve over time (Convention on Biological Diversity 2022; Obura et al. 2023; White et al. 2024; Bull et al. 2025)	Treated as a wider objective that usually depends on landscape-scale dynamics beyond facility control.
Intervention evidence	Verifiable records of actions implemented by the facility, including what was done, where, when, by whom, and how it is maintained (Boiral 2007; Boiral 2016; Joy-Camacho and Thornhill 2024)	The strongest NP4IF evidence layer because it is directly controllable, auditable, and linked to facility responsibilities.
Ecological signal	Observed change or trend in habitat condition, disturbance, species presence, or other ecological indicators (Mair et al. 2021; Xue et al. 2022; Yan et al. 2024; Wauchope et al. 2024)	Used as evidence for learning and adaptive management, not as definitive proof of net biodiversity gain.
Biodiversity claim	Statement made by a facility about its biodiversity actions, results, or contribution to nature-positive outcomes (Boiral 2016; Blanco-Zaitegi et al. 2022; Senanayake et al. 2024)	Must be calibrated to evidence strength: strong for implemented and maintained actions; cautious for ecological outcomes.
Net biodiversity gain	Claim that biodiversity gains exceed losses after applying baseline, equivalence, additionality, permanence, attribution, and governance criteria (Bull et al. 2013; Arlidge et al. 2018; Maron et al. 2024; Wauchope et al. 2024)	Not the default NP4IF claim because such evidence is difficult to establish credibly at facility scale.
Nature-positive	Directional ambition to halt and reverse biodiversity loss and improve nature (Locke et al. 2021; Milner-Gulland 2022; Obura et al. 2023; White et al. 2024; Buckley et al. 2025; Stenseke 2025)	Interpreted in NP4IF as a disciplined facility-scale stewardship process, not as automatic proof of net ecological recovery.

## Review Results

### Nature-Positive as a Contested Concept

The reviewed literature does not use nature-positive in a single, stable way. In its strongest and most widely cited sense, the term denotes a societal ambition to halt and reverse biodiversity loss and to move beyond damage limitation towards ecological recovery (Locke et al. 2021; Obura et al. 2023). In this usage, nature-positive functions as a directional goal across scales rather than as a directly verifiable site-level outcome. At the same time, several authors warn that the concept becomes analytically weak when it is applied without clear specification of scale, baseline, evidence standard, or governance mechanism (Milner-Gulland 2022; Thomas et al. 2024; White et al. 2024; Stenseke 2025). As a result, nature-positive may refer to a global objective, a corporate contribution, a project-level outcome, or a broad rhetorical signal of environmental intent (Krause et al. 2020; zu Ermgassen et al. 2022).

For industrial facilities, this ambiguity is especially problematic. A facility is a bounded operational site within a wider ecological landscape, not a biome, jurisdiction, or investment portfolio. When nature-positive is interpreted as a site-level net-outcome claim, unresolved questions immediately arise about what is being measured, against which baseline, over what time horizon, and with what degree of causal attribution. When it is used only as a motivational slogan, however, it offers little operational guidance. The literature therefore suggests that a credible facility-scale interpretation must distinguish directional ambition from outcome claims and translate nature-positive into a more bounded logic of biodiversity stewardship (Milner-Gulland 2022; Ferrier 2024; Bull et al. 2025).

### Netting, Compensation, and the Mitigation Hierarchy

A second recurring theme concerns the relationship between nature-positive and earlier net-based biodiversity frameworks, especially no net loss (NNL), net positive impact (NPI), biodiversity net gain (BNG), and the mitigation hierarchy (UK Government 2023). These approaches share a balancing logic in which residual losses are expected to be matched or exceeded by gains secured through restoration, rehabilitation, or compensation (Bull et al. 2013; Arlidge et al. 2018; Bull et al. 2020). Their appeal lies in their apparent compatibility with a familiar performance logic: define a baseline, calculate losses and gains, and report a net result.

The literature, however, identifies persistent integrity problems in this logic (Griffiths et al. 2019). The most recurrent concerns are equivalence, additionality, timing, and permanence. Ecological gains created through compensation may not be genuinely comparable to losses incurred elsewhere; some gains may have occurred without the intervention; restoration often takes much longer than the impact; and long-term maintenance remains uncertain under changing ownership, finance, or management conditions (Bull et al. 2013; Bull and Strange 2018; Carver 2021; Kujala et al. 2022; Cares et al. 2025). Recent contributions do not resolve these concerns. Rather, they reinforce the need for stricter mitigation-hierarchy discipline and caution against allowing nature-positive language to legitimise premature compensatory claims (Maron et al. 2024; Morrison-Saunders and Sánchez 2024; Wauchope et al. 2024; Duffus et al. 2025).

For industrial facilities, the implication is not that compensation is always irrelevant, but that it provides a fragile basis for general claims of positive biodiversity performance. The literature more strongly supports a default emphasis on on-site avoidance, pressure reduction, habitat improvement, and continuity of management than on abstract balancing claims.

### Facility-Scale Controllability and Attribution Limits

A third result concerns limited controllability. Across the reviewed literature, industrial managers are shown to exert direct influence over many proximate determinants of habitat condition, including mowing regimes, planting structure, drainage patterns, lighting, herbicide use, and contractor specifications. These interventions can meaningfully improve habitat quality within the site perimeter and therefore matter for facility-scale biodiversity stewardship (Rainey et al. 2015).

At the same time, managers cannot fully control ecological outcomes in the stronger sense implied by some nature-positive claims. Species occurrence, abundance, and persistence depend on landscape connectivity, source populations, climatic variation, predation, and ecological time lags beyond the facility boundary. Improved habitat may therefore be necessary for biodiversity recovery without being sufficient to guarantee it. This distinction has been highlighted in sector-specific work on mining and offshore wind, where guidance quickly encounters limits of causal attribution once site actions are translated into claims about biodiversity outcomes (Victurine et al. 2024; Margarido et al. 2025; Pardo et al. 2025).

This asymmetry is important in three ways. First, it affects accountability because managers can be held accountable for actions that are implemented, maintained, and governed, but not for every ecological response over longer time horizons. Second, it affects monitoring because ecological observations are better treated as directional learning signals than as deterministic performance scores. Third, it affects communication because stronger claims are justified for interventions and governance routines than for biodiversity outcomes themselves (Boiral 2016; Blanco-Zaitegi et al. 2022; zu Ermgassen et al. 2022).

### Measurement, Reporting, and Evidence Quality

The review also shows that biodiversity measurement is not only a technical problem but a conceptual one. Biodiversity is multidimensional, encompassing habitats, species, ecological functions, and relationships. Any metric therefore captures only selected aspects of ecological change. Different indicator choices can lead to different ecological and managerial conclusions, and no single metric can provide a complete representation of biodiversity condition (Nicholson et al. 2021; Martínez-Jauregui et al. 2021; Simpson et al. 2022; Marshall et al. 2024; Wauchope et al. 2024). This is especially important for industrial facilities, where measurement systems must often be feasible, repeatable, and proportionate rather than highly elaborate.

The reporting literature adds a second concern. Biodiversity disclosure is increasingly expected through instruments such as TNFD, ESRS E4, and science-based target initiatives, but accounting and reporting practices remain fragmented, selective, and vulnerable to impression management (Boiral 2016; Blanco-Zaitegi et al. 2022; European Union 2023; IFRS/ISSB 2023; TNFD 2023; Science Based Targets Network 2024; Senanayake et al. 2024). For facility management, the review therefore supports a two-layer evidence logic. The first layer is intervention evidence: records of what was implemented, where, when, under whose responsibility, and with what maintenance provisions. The second is ecological signal evidence: repeated observations of habitat condition or ecological response that are relevant for learning but should not be overstated as proof of net biodiversity outcomes (Mair et al. 2021; Xue et al. 2022; Yan et al. 2024).

### EMS Integration and Persistence

A final recurring theme is that biodiversity actions rarely persist unless they are embedded in routine management systems. The EMS literature shows that environmental management systems can function either as symbolic commitments or as practical infrastructures, depending on how deeply they are integrated into everyday routines, audits, work instructions, corrective actions, and management review (Boiral 2007). This insight is particularly relevant to biodiversity stewardship, which is vulnerable to reversal through procurement changes, contractor drift, budget cuts, and staff turnover. Similar challenges are evident in SMEs, where successful implementation depends on proportionality, organisational fit, and integration with existing management processes (Joy-Camacho and Thornhill 2024).

Across the reviewed literature, credible biodiversity stewardship at the facility scale is therefore associated less with ambitious rhetoric than with mundane but durable mechanisms: site zoning, maintenance standards, contractor clauses, inspection schedules, monitoring sheets, escalation procedures, and periodic review. For many facilities, especially SMEs and legacy sites, a limited but persistent stewardship system may be more defensible than expansive claims unsupported by operational continuity.

### Synthesis of Review Results

The review identifies four recurrent tensions shaping biodiversity stewardship at industrial facilities. The first is semantic because nature-positive is used both as a societal ambition and as a site-level claim. The second is methodological because netting and offsetting remain fragile where biodiversity units, equivalence, and permanence are disputed. The third is managerial because facilities control interventions more directly than outcomes. The fourth is institutional because biodiversity actions often remain weak unless they are embedded in EMS-type routines and persistence mechanisms (Ferrier 2024; Morrison-Saunders and Sánchez 2024; White et al. 2024; Bull et al. 2025).

We infer from these findings that facility-scale biodiversity stewardship is most credible when it is local, pressure-oriented, operationally embedded, and calibrated to evidence quality and controllability. This synthesis provides the analytical basis from which we develop NP4IF as an environmental management framework in Section 5.

## Discussion

The review indicates that the main challenge in applying nature-positive to industrial facilities is not only conceptual ambiguity, but also environmental management feasibility. At the global scale, nature-positive can function as a mobilising goal for halting and reversing biodiversity loss (Locke et al. 2021; Obura et al. 2023). At the facility scale, however, it becomes credible only when translated into a bounded stewardship logic tied to controllable interventions, operational routines, and cautious claims (Milner-Gulland 2022; White et al. 2024; Stenseke 2025). This scalar distinction helps to reconcile two strands in the literature: one promoting nature-positive as a societal ambition, and another warning that the term loses clarity when transferred uncritically to project-, firm-, or site-level claims (Locke et al. 2021; Milner-Gulland 2022; White et al. 2024; Ferrier 2024; Bull et al. 2025).

A second implication concerns accountability. Much biodiversity discourse still reflects an accounting logic in which responsibility is demonstrated through standardised indicators and net outcomes. The review suggests that this model fits industrial facilities only weakly. At the site level, accountability is more defensible when attached to actions that have been implemented and maintained than to biodiversity outcome claims that exceed managerial control (Boiral 2016; Blanco-Zaitegi et al. 2022; Senanayake et al. 2024). This is consistent with wider critiques of netting and compensation, which continue to identify unresolved problems of equivalence, additionality, timing, permanence, and governance (Bull et al. 2013; Arlidge et al. 2018; Maron et al. 2024; Morrison-Saunders and Sánchez 2024; Wauchope et al. 2024). Accordingly, stronger claims should concern reduced pressures, implemented interventions, and embedded routines, while ecological observations are better treated as signals for learning than as definitive proof of net biodiversity success (Mair et al. 2021; Xue et al. 2022; Yan et al. 2024).

These findings have practical implications for industrial facilities, especially SMEs and legacy sites. A credible approach begins with a bounded operational perimeter, a simple baseline focused on habitat condition and controllable pressures, and a limited portfolio of feasible actions such as mowing changes, planting diversification, drainage enhancement, lighting attenuation, or reduced chemical use. These actions must then be embedded in routine management systems through work instructions, contractor clauses, inspections, corrective actions, and periodic review (Boiral 2007; Joy-Camacho and Thornhill 2024). This is important because many facilities lack the resources for elaborate biodiversity accounting, but can still adopt a proportionate and persistent stewardship system.

The review also has wider international relevance. Although biodiversity regulation and reporting differ across jurisdictions, industrial facilities in many countries face similar operational conditions: bounded sites, legacy infrastructure, maintenance contracts, safety constraints, and uneven ecological capability. For this reason, the review contributes not a jurisdiction-specific model, but a transferable logic of facility-scale stewardship: local, non-compensatory by default, operationally embedded, and explicit about uncertainty. Its main strength lies in integrating biodiversity governance, conservation, reporting, and EMS literatures; its main limitation is that it is structured and critical rather than fully systematic and is weighted towards Anglophone and European debates. Even so, the synthesis helps to clarify how high-level biodiversity ambition can be translated into more credible environmental management practice.

Based on the reviewed literature, we propose shifting from broad nature-positive rhetoric towards a bounded management logic for industrial facilities. As an implication for practice, the relevant question is whether facilities can improve habitat condition and ecological function through controllable and persistent actions, rather than whether they can routinely demonstrate universal net biodiversity success.

## NP4IF as an Environmental Management Framework

NP4IF is proposed as a facility-scale environmental management framework for biodiversity stewardship. It translates broad nature-positive ambition into a bounded management process that can be implemented, monitored, reviewed, and communicated at the level of an industrial site. NP4IF is not a new biodiversity doctrine or a substitute for wider nature-positive, no net loss, biodiversity net gain, TNFD, ESRS, or science-based target approaches. Its contribution is operational: it specifies how biodiversity improvement can be organised through the spaces, routines, constraints, and responsibilities that facility managers can actually influence.

The novelty of NP4IF lies in combining six elements that are usually treated separately in the literature: the facility perimeter as the unit of action and claim; a controllability filter; prioritisation of feasible interventions; EMS-compatible governance; two-layer evidence; and disciplined biodiversity claims. This responds to the instability of nature-positive terminology and to the methodological limits of site-level net-outcome claims (Milner-Gulland 2022; White et al. 2024; Buckley et al. 2025; Stenseke 2025). It also reflects long-standing concerns about equivalence, additionality, timing, permanence, and governance in offsetting and net-gain approaches (Bull et al. 2013; Maron et al. 2016; Bull and Strange 2018; Bull et al. 2020; Kujala et al. 2022; Wauchope et al. 2024).

### Framework Components

NP4IF is composed of seven connected components. First, the facility perimeter defines the default unit of action, evidence, responsibility, and claim. Second, a baseline identifies relevant ecological zones, habitat condition, operational constraints, and controllable pressures. Third, a prioritisation filter selects interventions according to ecological relevance, feasibility, safety, cost, persistence, and the mitigation hierarchy. Fourth, an intervention portfolio translates priorities into practical actions, such as vegetation management, planting diversification, drainage improvement, lighting attenuation, disturbance reduction, and reduced chemical pressure (Rainey et al. 2015; Science Based Targets Network 2024). Fifth, governance mechanisms embed actions into routine operations through responsibilities, work instructions, contractor clauses, maintenance plans, inspections, corrective actions, and management review. Sixth, a two-layer evidence system distinguishes between intervention evidence, which is controllable and auditable, and ecological signal evidence, which is useful for learning but must be interpreted cautiously (Mair et al. 2021; Xue et al. 2022; Yan et al. 2024). Seventh, a claims-discipline mechanism aligns external communication with the strength of evidence, reducing the risk of overstatement or impression management (Boiral 2016; Blanco-Zaitegi et al. 2022; Senanayake et al. 2024).

### Relationships Among Components

The components of NP4IF are sequential but iterative. The facility perimeter determines the scope of the baseline. The baseline identifies pressures and opportunities. The prioritisation filter converts these into a limited intervention portfolio. Governance mechanisms ensure that the portfolio is not treated as a one-off project but as part of the facility’s operating system. Monitoring then generates two forms of evidence: records of what has been implemented and ecological observations that may indicate change. Finally, claims discipline determines what can be communicated with confidence and what must remain qualified.

This relationship is important because industrial facilities control management actions more directly than biodiversity outcomes. Managers can modify habitat condition and reduce pressures, but they cannot fully control species occurrence, population persistence, or long-term ecological recovery, which also depend on landscape context, time lags, and wider ecological dynamics (Victurine et al. 2024; Pardo et al. 2025). NP4IF therefore links accountability to controllable actions while treating ecological outcomes as evidence for learning rather than automatic proof of net biodiversity gain.

### Implementation Stages

**Fig. 2:**
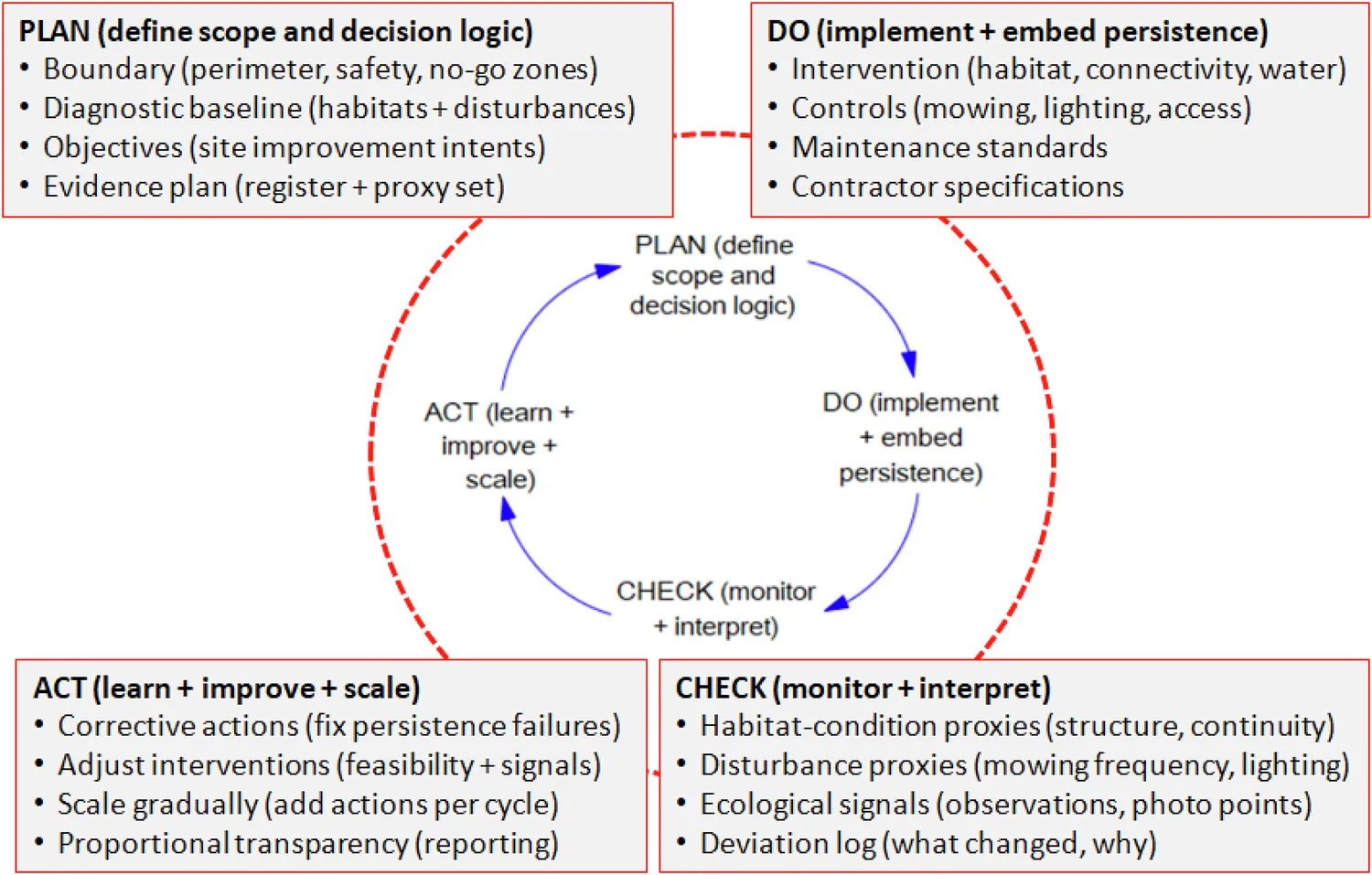
NP4IF facility-scale implementation cycle: a PDCA-compatible stewardship loop. Source: authors’ elaboration based on EMS/PDCA logic and the two-layer evidence model, drawing on Boiral (2007), Boiral (2016), Joy-Camacho and Thornhill (2024), Mair et al. (2021), Xue et al. (2022), and Yan et al. (2024)

NP4IF follows a PDCA-compatible implementation sequence (Fig. 2). In the Plan stage, the facility defines the perimeter, identifies ecological zones, reviews legal and operational constraints, establishes a simple baseline, and selects a small set of feasible priorities. In the Do stage, selected interventions are implemented through operational controls, SOPs, maintenance routines, contractor requirements, training, and responsibility assignment. In the Check stage, the facility verifies intervention continuity and records ecological observations using proportionate indicators. In the Act stage, the facility corrects weaknesses, adapts measures, scales effective actions, discontinues ineffective measures, and updates management review.

This staged logic connects biodiversity stewardship with the practical infrastructure of environmental management systems. EMS routines can support implementation, but they do not by themselves define biodiversity-specific evidence standards, ecological priorities, or claim boundaries. NP4IF adds this biodiversity-specific operational layer to EMS practice (Boiral 2007; Boiral 2016; Joy-Camacho and Thornhill 2024).

### Decision Pathway and Claims Discipline

As a proposed management recommendation, NP4IF uses a decision pathway to reduce the risk of symbolic implementation or overstated biodiversity claims.

- Point 1. Is the issue within the facility perimeter or under direct facility influence? If yes, continue to Point 2. If not, treat it as an external contribution or partnership action, not as core facility-level NP4IF evidence.
- Point 2. Can the action comply with legal, safety, hygiene, operational, and production requirements? If yes, continue to Point 3. If not, redesign, relocate, postpone, or exclude the action.
- Point 3. Can the pressure first be avoided or reduced? If yes, prioritise avoidance or reduction measures and continue to Point 4. If not, consider habitat improvement only if it is feasible, relevant, and not presented as compensation for unresolved impacts.
- Point 4. Can the intervention be maintained through routine operations? If yes, continue to Point 5. If not, do not prioritise the intervention unless continuity can be secured.
- Point 5. Can implementation be recorded, verified, and reviewed? If yes, include it in the intervention evidence layer and continue to Point 6. If not, do not use it to support strong NP4IF claims.
- Point 6. Are ecological observations or monitoring results available? If yes, report them as ecological signals or trends, with uncertainty. If not, report only the implemented and maintained actions, and avoid claims of demonstrated ecological improvement.

Within NP4IF, this pathway is used to determine the level of claim that can be justified by the available evidence. NP4IF recommends that strong claims be limited to actions that have been implemented, maintained, and governed. Qualified claims may concern ecological signals or trends. NP4IF recommends avoiding claims of net biodiversity gain or of a facility being definitively nature-positive unless robust evidence exists for baseline, equivalence, additionality, permanence, attribution, and governance (Bull et al. 2013; Arlidge et al. 2018; Maron et al. 2024; Wauchope et al. 2024).

Table 2 translates the NP4IF framework into actions for industrial facilities. Each stage links a management question to the corresponding actions, evidence requirements, and EMS linkage. Its purpose is not to demonstrate facility-level net biodiversity gain, but to define a disciplined pathway through which controllable interventions can be planned, implemented, monitored, reviewed, and communicated. The distinction between intervention evidence and ecological signal evidence is central: facilities can be held directly accountable for actions that are implemented, maintained, and governed within their perimeter, whereas ecological observations should be interpreted cautiously because biodiversity outcomes also depend on wider landscape conditions, temporal dynamics, and partial controllability.

**Table 2:** NP4IF implementation matrix for facility-scale biodiversity stewardship

NP4IF stage	Management question	Main actions	Evidence generated	EMS link
Site assessment	What is within facility control?	Define perimeter, zones, baseline, constraints	Site map, baseline, pressures	Aspects/impacts review
Priority	Which interventions are credible and feasible?	Select actions using controllability, mitigation hierarchy, safety, feasibility	Action plan, priority rationale	Objectives and planning
Implement	How are actions embedded?	SOPs, maintenance, contractor clauses, responsibilities	Intervention records	Operational control
Monitoring	What changed and what is uncertain?	Intervention checks and ecological observations	Two-layer evidence	Monitoring and evaluation
Governance review	Are actions persistent and improving?	Audits, corrective actions, management review	Audit trail, corrective actions	Internal audit and management review
Reporting	What can be communicated credibly?	Align claims with evidence strength	Disclosure note, uncertainty statement	External reporting support

### Scope and Limits of NP4IF

NP4IF applies primarily to industrial facilities where managers can influence land, vegetation, drainage, lighting, disturbance, chemical use, maintenance routines, contractors, and local habitat condition. It is especially relevant for manufacturing sites, logistics centres, energy facilities, and SMEs where biodiversity action must be feasible, proportionate, and operationally embedded. It is less suitable as a stand-alone framework for landscape-scale restoration, protected-area management, biodiversity markets, or corporate-level net accounting.

NP4IF does not replace legal compliance, pollution prevention, climate mitigation, environmental impact assessment, TNFD, ESRS E4, SBTN, or biodiversity net gain requirements. Rather, it provides the implementation layer through which high-level biodiversity expectations can be translated into practical facility management. Its limits are also its safeguard: it does not promise full ecological recovery, guaranteed species return, or universal net gain. It provides a disciplined method for improving local habitat condition and reducing controllable pressures while communicating results with appropriate caution (TNFD 2023; European Union 2023; Science Based Targets Network 2024; Bull et al. 2025).

NP4IF should therefore be understood as compatible with, but narrower than, ecosystem recovery approaches. Recent nature-positive and biodiversity-policy debates increasingly emphasise ecosystem condition, ecological integrity, resilience, and recovery of natural processes (Convention on Biological Diversity 2022; Obura et al. 2023; Ferrier 2024; White et al. 2024; Bull et al. 2025). Industrial facilities can contribute to these wider aims by improving habitat condition, reducing controllable pressures, and maintaining interventions over time. However, ecosystem recovery usually depends on larger spatial scales, landscape connectivity, species source populations, and long ecological timeframes that exceed the control of a single facility. NP4IF therefore treats ecosystem recovery as a broader reference objective, while defining facility-level contribution in terms of controllable actions, habitat-condition improvement, ecological signals, and cautious claims.

Facilities with low ecological potential may contribute through landscape restoration partnerships, catchment initiatives, supply-chain biodiversity requirements, or carefully governed biodiversity credit schemes. These should be reported separately from core NP4IF evidence unless they are directly linked to facility-controlled action and robust governance (Bull et al. 2020; Damiens et al. 2021; Maron et al. 2024; Morrison-Saunders and Sánchez 2024; and Wauchope et al. 2024).

## Future Research

The review points to several priorities for future research. First, more evidence is needed on the persistence of facility-scale biodiversity actions: which interventions endure changes in budgets, contractors, retrofits, and management attention, and which decline once symbolic commitment fades. Longitudinal studies tracking the same facilities over time would be particularly valuable.

Second, further work is needed on indicators and evidence standards. Research should test which small, feasible indicator sets best capture changes in habitat condition and ecological function without encouraging false precision. This includes examining how photographic records, simple field observations, habitat-condition scores, and repeat monitoring can be combined in ways that remain useful for management and proportionate for industrial sites (Mair et al. 2021; Xue et al. 2022; Yan et al. 2024).

Third, the literature needs a stronger empirical basis on adoption pathways, especially in SMEs and legacy industrial facilities. Relevant questions include how firms develop ecological capability, how they rely on external advisers, what barriers arise in procurement and maintenance systems, and which support mechanisms most effectively stabilise implementation (Joy-Camacho and Thornhill 2024).

Fourth, more research is needed on EMS integration. Existing EMS literature offers useful guidance, but more evidence is required on how biodiversity-specific routines interact with audits, contractor management, corrective actions, and management review across different sectors (Boiral 2007). Finally, future work should examine the relationship between site-level evidence and external disclosure, especially as TNFD, ESRS, and science-based target initiatives continue to develop. A key question is whether disclosure strengthens substantive stewardship or mainly encourages more sophisticated claims (TNFD 2023; European Union 2023; Science Based Targets Network 2024; zu Ermgassen et al. 2022).

## Conclusions

This article examined how nature-positive can be translated into credible environmental management practice at the scale of industrial facilities. The review shows that nature-positive remains influential but unstable, especially when a broad societal ambition is converted into a facility-level claim without clear specification of scale, baseline, evidence, attribution, and governance (Locke et al. 2021; Milner-Gulland 2022; Obura et al. 2023; White et al. 2024; Buckley et al. 2025; Stenseke 2025).

The findings identify four main constraints on facility-scale biodiversity stewardship: ambiguity between nature-positive as a global goal and as a site-level claim; methodological fragility in net-based approaches where equivalence, additionality, timing, permanence, and governance remain contested; limited managerial control over ecological outcomes; and weak persistence when biodiversity actions are not embedded in operational routines (Bull et al. 2013; Arlidge et al. 2018; Bull et al. 2020; Boiral 2007; Boiral 2016; Maron et al. 2024; Morrison-Saunders and Sánchez 2024; Wauchope et al. 2024).

On this basis, the article proposes Nature-Positive for Industrial Facilities (NP4IF) as a facility-scale implementation architecture. Its contribution is to connect biodiversity stewardship to the industrial facility perimeter as the unit of action, evidence, responsibility, and claim. NP4IF links site assessment, prioritisation of controllable interventions, operational integration, two-layer monitoring, governance review, reporting discipline, and adaptive management. In this sense, it complements wider biodiversity governance, disclosure, and target-setting frameworks rather than replacing them (Convention on Biological Diversity 2022; TNFD 2023; European Union 2023; Science Based Targets Network 2024).

The central implication is that industrial facilities should avoid premature claims that they are definitively “nature-positive” in abstract net-outcome terms. A more credible claim is that a facility has implemented, maintained, monitored, and reviewed a structured biodiversity stewardship process within its perimeter, while reporting ecological observations as signals or trends rather than as definitive proof of net biodiversity gain. NP4IF therefore supports a shift from nature-positive as a broad label to nature-positive as a disciplined management practice: local, controllable, persistent, evidence-based, and communicated with appropriate caution (Nicholson et al. 2021; Blanco-Zaitegi et al. 2022; Senanayake et al. 2024; Bull et al. 2025).

## Supplementary information

Supplementary material

## Data Availability

No datasets were generated or analysed during the current study.
