# Supplementary material for: Nature-Positive for Industrial Facilities: An Environmental Management Framework for Biodiversity Stewardship
Source: Environ Manage. 2026 Sep 1;76(9):298. doi: 10.1007/s00267-026-02601-2 (PMC13534218; doi:10.1007/s00267-026-02601-2)
Supplement: Supplementary file 1 [file 267_2026_2601_MOESM1_ESM.docx]

Nature-Positive for Industrial Facilities: An Environmental Management Framework for Biodiversity Stewardship

Ángela Martín-Méndez ^[1]^ - Francisco Campuzano-Bolarín ^[2]^ ^(c)^ - David López-López^[3]^

**Supplementary Materials**

Content

[SM 1. Positioning N-P among adjacent frameworks	2](#_Toc223006338)

[SM 2. Sustainability vs. Nature-Positive	6](#_Toc223006339)

[SM 3. A practical implementation model for SMEs	15](#_Toc223006340)

[SM 4. Menu of actions	18](#_Toc223006341)

[SM 5. Monitoring templates	26](#_Toc223006342)

[SM 6. Examples	31](#_Toc223006343)

## SM 1. Positioning N-P among adjacent frameworks

Industrial Nature-Positive (N-P) is best positioned as an implementation backbone: it translates biodiversity ambition into facility-scale design requirements, operational controls, monitoring routines, and stewardship continuity. Adjacent frameworks often excel at direction-setting (policy), target-setting (science alignment), disclosure (comparability and accountability), or project compliance (planning instruments). N-P is distinct because it operates where ecological outcomes are actually produced: the managed spaces, disturbances, and maintenance practices of industrial sites.

Across these frameworks a consistent pattern emerges: disclosure systems (e.g., ESRS, TNFD, IFRS/ISSB) structure transparency and risk communication; policy agendas (e.g., SDGs, KMGBF) provide directionality; planning instruments (e.g., BNG) enforce improvement in defined contexts; and conservation institutions (e.g., IUCN) provide principles and review logic. N-P is distinct because it targets the point of production of outcomes, industrial site design and management, thereby making adjacent frameworks more implementable, auditable, and less vulnerable to symbolic compliance.

**1.1 ESG**

ESG is primarily an evaluative and capital-market interface: it reduces multi-dimensional environmental and social realities to comparable signals for governance, valuation, and stakeholder engagement. Even when biodiversity is covered, ESG typically rewards policies, risk management, and disclosure quality more readily than durable site outcomes. N-P complements ESG by supplying decision-grade, facility-anchored evidence (interventions, maintenance controls, and ecological signals) that reduces reliance on narrative proxies and helps distinguish ecological performance from management pressures.

**1.2. NNL+NPI**

Nature-Positive (N-P), as defined in this study, differs fundamentally from No Net Loss (NNL) and Net Positive Impact (NPI) in its conceptual foundation and practical implications. NNL and NPI are built on an accounting logic. NNL seeks to ensure that biodiversity losses are fully balanced, while NPI aims to demonstrate a measurable net gain. Both approaches depend on baselines, quantification, equivalence between losses and gains, and often compensatory measures. Their implementation requires robust metrics, long-term monitoring and defensible calculations of ecological balance. This makes them technically demanding, costly and conceptually difficult, particularly because biodiversity is dynamic, context-dependent and not easily reducible to comparable units. Industrial N-P departs from this accounting paradigm. It is grounded in facility-scale stewardship and focuses on sustained ecological improvement within the operational perimeter of a site. Rather than claiming balance or surplus, it centres accountability on controllable interventions and their persistence over time. Ecological outcomes are interpreted as directional signals under uncertainty, not as precise net calculations. In short, NNL and NPI rely on proving ecological balance or gain through measurement and equivalence, which makes them difficult to implement in practice. Industrial N-P lowers the evidentiary threshold by concentrating on what facilities can directly manage, shifting from biodiversity accounting to operational stewardship.

**1.3 BNG**

BNG, used by the UK Environment Act 2021, is a planning and development centered instrument requiring measurable improvement relative to a pre-development baseline, with statutory expectations (e.g., a minimum percentage gain) and long-term securing of outcomes through legal mechanisms in the English system. N-P aligns in ambition moving beyond NNL toward improvement, but differs in system boundary and life-cycle logic: N-P treats industrial assets as enduring infrastructures where biodiversity outcomes depend on design, operations, and maintenance continuity across decades. N-P can use BNG-style metrics where appropriate, while adding the operational machinery needed to avoid “metric-true but ecology-weak” outcomes driven by time lags, spatial mismatch, or management failure.

**1.4 CSRD and ESRS E4**

CSRD and ESRS E4 specify what must be disclosed and how, under a double-materiality logic, about impacts, dependencies, risks, and opportunities related to biodiversity and ecosystems. Their function is governance through transparency and comparability; they do not constitute a method for producing ecological performance. N-P complements them by providing a coherent evidence chain—baseline description, intervention logic, monitoring protocols, and assurance-ready documentation—that can populate disclosures with traceable operational content. In this way, ESRS can compel transparency, while N-P makes transparency meaningful by anchoring it in facility practice.

**1.5 TNFD and LEAP**

TNFD structures identification, assessment, management, and disclosure of nature-related dependencies, impacts, risks, and opportunities, with LEAP providing a practical sequence for analysis. Its comparative advantage is in framing risk and disclosure coherently for markets and governance. N-P complements TNFD by translating “Locate and Evaluate” into facility-relevant baselines and by converting “Assess and Prepare” into concrete design standards, operating rules, and monitoring routines. Used together, TNFD can structure why and what should be managed and disclosed, while N-P specifies how ecological performance is delivered at the site.

**1.6 SBTN and SBTs for Nature**

SBTN provides methodologies for science-based targets across nature domains (e.g., freshwater and land), treating biodiversity as cross-cutting. Its strength is translating scientific constraints into ambition levels and target-setting discipline; its limitation is that it does not, by itself, determine how an industrial facility should be configured operationally to meet targets. N-P supplies the implementation grammar: it converts target intent into intervention portfolios, maintenance standards, disturbance controls, and feasible proxy monitoring suitable for industrial constraints and small and medium enterprises.

**1.7 KMGBF Target 15**

Target 15 strengthens the global expectation that companies—especially large and transnational firms and financial institutions—monitor, assess, and disclose biodiversity-related dependencies and impacts. This increases institutional pressure for disclosure and impact management, but it does not prescribe a facility-scale method for producing local biodiversity improvement. N-P complements the policy direction by enabling a shift from “assess and disclose” to “design and deliver,” reducing the risk that global ambition becomes primarily an accounting exercise.

**1.8 SDGs**

The SDGs provide normative directionality across social, economic, and environmental dimensions, and in industry often translate into mapping exercises, high-level targets, and contribution narratives. N-P complements this agenda by converting broad alignment into measurable site-level contributions, particularly for SDG 15 and SDG 14, and indirectly for goals linked to water, climate, cities, consumption, and infrastructure. The practical advantage is that N-P can anchor SDG claims in verifiable facility actions and ecological signals rather than generalized statements of support.

**1.9 IUCN net gain and net positive approaches**

IUCN policy and review tools emphasize that net outcomes should be achieved “on the ground” and governed through credible process and evidence, grounded in mitigation hierarchy discipline. N-P aligns with this family of approaches but differs in how it is managed: it treats industrial assets as engineered systems with long lifetimes, safety constraints, recurring disturbances, and frequent retrofit cycles. The added value is to embed net gain intent into industrial standards, operating controls, and assurance routines that support persistence.

**1.10 IFRS/ISSB sustainability disclosure standards**

IFRS S1 and IFRS S2 institutionalize sustainability-related financial disclosure globally (with climate specificity in S2), reinforcing the mainstreaming of disclosure as a governance mechanism. These standards strengthen comparability and reporting discipline but do not provide an operational method for producing ecological improvement at facilities. N-P remains distinct because it is not a disclosure standard; it is the facility-scale practice that can populate disclosures with robust evidence and reduce the gap between reported intent and ecological effect.

**1.11 UK Environment Act 2021 and the planning pull**

The Environment Act 2021 and related mechanisms (including local nature recovery strategies and BNG conditions in the English planning context) illustrate a broader regulatory trend: biodiversity outcomes (in fact only the habitat) increasingly shift from “good practice” to enforceable expectations in defined domains. For industrial sites, this creates a compliance and capability challenge, particularly during expansions and retrofits. N-P complements the regulatory pull by making biodiversity improvement implementable within industrial facilities and by reducing the tendency to treat compliance as a one-off hurdle.

Figure 4: Main screen of the Excel file to calculate the net increase in biodiversity.

UK Environment Act 2021 has two basic aspects: its mandatory nature regarding Biodiversity Net Gain (BNG), and its global approach, which allows for offsetting losses in one location with gains in another, since it is generally not possible to achieve a net gain where the industry is located. In contrast, the N-P approach presented here is local, and regardless of compliance with mandatory legal requirements, it offers guidance to industries to improve biodiversity within the factory perimeter where possible (Figure 4). Therefore, it is not mandatory (since it is usually not possible locally) nor does it allow for offsets (typical of a global approach like BNG).

**1.12 Biodiversity credits, offsets, and habitat banks**

Market instruments can mobilize finance toward biodiversity outcomes, but the literature highlights persistent concerns regarding equivalence, permanence, and verification, making integrity highly sensitive to governance design. N-P does not need to reject these instruments categorically; instead, it shifts the default priority toward on-site and landscape-integrated performance first, treating market instruments as secondary mechanisms requiring strong safeguards. This positioning also aligns with the non-compensatory logic advanced for industrial facilities, where local impacts should be managed locally whenever feasible.

## SM 2. Sustainability vs. Nature-Positive

**A bounded definition of “nature” for industrial use**

Because N-P is used inconsistently, industrial implementation requires a bounded operational definition that can guide intervention design and discipline claims. In this study, “nature” is equated with biodiversity, but biodiversity is managed through two linked domains with different levels of managerial influence: habitat condition and ecological function, which depend directly on facility practices, and fauna outcomes, which are only partially dependent and should therefore be treated as contingent signals rather than guaranteed results (Bull et al., 2020; de Silva et al., 2019). This framing aligns with the mitigation hierarchy (MCH) emphasis that N-P must incorporate rather than undermine avoidance, minimisation and restoration priorities, because these are the levers most compatible with facility-scale control (Arlidge et al., 2018; Maron et al., 2024).

Habitat condition are defined here as the ecological quality of spaces within the facility perimeter, including vegetation structure and diversity, soil condition, microhabitats, hydrological features, and the disturbance regime created by operations and maintenance. These elements can be planned, implemented, and maintained, and they can be evidenced through auditable records and monitoring. Fauna outcomes, by contrast, are influenced by habitat but are also constrained by exogenous factors.

The definition has direct implications. First, it discourages translating biodiversity into a single unit for corporate “netting”, a move that is deeply contested (Carver, 2021; Bull et al., 2013). Second, it supports a two-layer (SM3) logic in which evidence is anchored in implemented interventions and their persistence, while ecological signals (including fauna observations) are treated as learning inputs that may strengthen confidence over time but should not be over-interpreted (Boiral, 2016; Blanco-Zaitegi et al., 2022). Third, it reduces the risk that N-P becomes primarily rhetorical by forcing explicit statements about scope, controllability, and uncertainty in external communication (Buckley et al., 2025; Boiral, 2016).

Table 2: Details of the conceptual and practical differences in the industrial field.

|  | **Sustainability** | **Nature-Positive (NP4IF)** |
| --- | --- | --- |
| **Goal** | **Human.** Seeks to balance environmental protection with social welfare and economic performance over long time horizons, often framed through the Triple Bottom Line (TBL/3BL) and the United Nations Sustainable Development Goals (SDGs). The goal is expressed as reducing aggregate harm and enabling long-term human prosperity under planetary constraints. | **Biodiversity**. Seeks to improve the ecological condition of nature within the industrial facility perimeter**,** with local biodiversity and habitat as the primary objective. It is aligned with “net gain” aspirations but expressed as site-based ecological improvement rather than a broad balancing of multiple pillars. |
| **Focus** | **Prioritizes human welfare,** economic development, and risk management; environment is protected because ecological degradation affect long-term social and economic stability. In business, nature is integrated into Corporate Social Responsibility (CSR) and Environmental, Social and Governance (ESG) performance in which biodiversity is one issue among many. | **Prioritizes local biodiversity** and the wellbeing of local ecosystems as an end in itself at the site scale. N-P is treated as an specific objective for the facility, not merged with other corporate economic or social themes. |
| **Scope** | **Global.** Tends to be global and standardized, relying on common frameworks and comparable reporting architectures across countries (e.g., UN and EU policy and reporting schemes), with climate governance anchored in the United Nations Framework Convention on Climate Change (UNFCCC). | **Local.** Tends to be local and landscape-specific, tailored to the ecological context, industrial constraints, and implementation capacity of the site. It aligns conceptually with national and global biodiversity directionality (e.g., Kunming–Montreal Global Biodiversity Framework (KMGBF) and National Biodiversity Strategies and Action Plans (NBSAPs)) but remains implemented at facility scale. |
| **Baseline** | **Standards.** Benchmarks performance against current standards, targets, or peer expectations rather than an ecological before state. Baselines are often defined as *current performance vs. required trajectory* under Science-Based Targets (SBTs) and related target frameworks. | **Current** (or year 2020). Uses a local ecological status prior to improvement actions mainly as a diagnostic reference rather than as a strict accounting anchor. Baseline functions as starting conditions for management and may be informed by Locate, Evaluate, Assess and Prepare (LEAP) thinking and screening tools such as the Integrated Biodiversity Assessment Tool (IBAT). |
| **Nature strategy** | Emphasizes **prevention and mitigation** of impacts, often consistent with the Mitigation and Conservation Hierarchy (MCH): avoid, minimize, restore, and offset/compensate residual impacts. Practice communities such as the Business and Biodiversity Offsetting Programme (BBOP) and policy groups such as the Impact Mitigation and Ecological Compensation Thematic Group (IMEC) reflect this mitigation orientation. | Emphasizes **direct on-site improvement** of habitat condition and ecological function, and rejects elsewhere compensation as the default response to local biodiversity impacts. Where project planning tools (e.g., Biodiversity Net Gain (BNG)) are relevant, they are treated as complementary inputs, but the industrial N-P logic remains anchored in local action and stewardship. |
| **Impact domain** | Addresses **global externalities** and multi-domain impacts, including climate change, air and water pollution, resource use, and social value creation. Corporate climate reporting and risk assessment is commonly structured through Greenhouse Gas (GHG) accounting and Task Force on Climate-related Financial Disclosures (TCFD) approaches. | Focuses on **local** ecological impacts that facilities can plausibly influence: habitats, vegetation structure, insects and other invertebrates, and small birds and mammals, plus the integrity of site-level ecological processes (e.g., connectivity, disturbance regimes). It aligns conceptually with biodiversity science-policy and conservation institutions such as the Convention on Biological Diversity (CBD), the International Union for Conservation of Nature (IUCN), and the Intergovernmental Science-Policy Platform on Biodiversity and Ecosystem Services (IPBES). |
| **Metrics** | Relies on **standardized**, quantifiable key performance indicators (KPIs) (e.g., emissions, energy use, water, waste), enabling external comparability and assurance. Reporting ecosystems include the Corporate Sustainability Reporting Directive (CSRD), Global Reporting Initiative (GRI), and global financial disclosure standards from the International Sustainability Standards Board (ISSB) within the International Financial Reporting Standards (IFRS) architecture, alongside climate risk frameworks such as TCFD. | Relies on periodic direct **observations** and context-specific indicators as proxies for ecological condition because biodiversity measurement remains contested and value-laden. Evidence is often grounded in repeatable observations, habitat-condition indicators, and disturbance metrics, and may be aligned with Taskforce on Nature-related Financial Disclosures (TNFD) framing where useful, while recognizing that proxy-based monitoring cannot provide the same comparability as conventional KPIs. |
| **Measure-ment purpose** | **External.** Designed for external accountability: benchmarking, regulatory compliance, investor communication, and reputational signaling. ESG and CSRD-linked disclosure demands encourage comparability, which in turn incentivizes standardized metrics and verification. | **Internal.** Designed primarily for internal managerial control: selecting interventions, tracking operational performance, and learning what works at the site. Indicators are chosen for feasibility and decision value rather than comparability across firms, with transparency optional. |
| **Time horizon** | Operates through **long-term roadmaps**, compliance programs, and target trajectories, frequently linked to national policy commitments such as Nationally Determined Contributions (NDC) under the Paris climate regime and target-setting logics that emphasize Specific, Measurable, Achievable, Relevant and Timely (SMART) objectives. | Operates through **rapid initiation of actions** and adaptive improvement: start early, implement feasible measures, monitor proxies, and adjust. Time is treated less as a fixed compliance horizon and more as an iterative learning loop for site stewardship, consistent with the “do now, improve iteratively” logic commonly associated with net gain practice communities. |
| **Operations** | Frequently **shapes processes**, product design, resource efficiency, waste strategies, recycling, and circularity. Typical levers include circular economy “R-Strategies,” technology transitions such as Carbon Capture and Storage (CCS), and shifts in mobility and logistics including Alternative Fuel Vehicles (AFV). | **Avoids interference** with production and product characteristics. Operational levers are primarily located in the managed external environment of facilities: grounds management, lighting, drainage, connectivity, disturbance reduction, and in Nature-Based Solutions (NBS) that can be embedded around infrastructure without disrupting production. |
| **Business impact** | It often **involves high costs**, investments, and strategic repositioning, including process redesign, reporting system investments, and compliance burdens. Sustainability is therefore tied to financing, procurement expectations, and market access, and it frequently becomes a strategic management domain. | Designed to be **low relative cost** and low disruption, enabling uptake by SMEs and legacy facilities. Business impact is framed as incremental operational improvement and reputational benefit, not product or process transformation. |
| **Leadership** | Commonly led by **sustainability specialists**, regulatory experts, and consultants who navigate reporting standards, compliance obligations, and stakeholder expectations. In mature firms, environment is often institutionalized within Health, Safety and Environment (HSE) functions aligned with compliance. | Led by operations and **facility management teams** because the levers of change are operational (maintenance, disturbance regimes, site design and grounds management), with targeted support from biologists and local naturalists when needed. Leadership is oriented toward implementation feasibility and continuity. |
| **Stakehol-ders** | Typically requires strong external engagement: **regulators,** communities, non-governmental organizations (NGOs), and investors. Community engagement and consent processes may invoke Free, Prior and Informed Consent (FPIC) principles. | Focused on **facility-level** governance: seeking acceptance from employees and local communities. External involvement is proportional to local ecological and social relevance. |
| **Governance** | Driven by law, **regulation**, and supervisory enforcement; compliance is mandatory and failure can produce penalties. In Europe, sustainability governance increasingly couples reporting and due diligence under EU policy, with CSRD and related standards shaping corporate accountability. | Governed as **voluntary** guidance at facility level, with actions potentially reversible if production needs change. This is not an omission of accountability, but rather a feature to make it viable under conditions of uncertainty and for its adoption by SMEs that lack the capacity to obtain a guarantee of results. |
| **Offsetting** | Accepts that some global impacts **can be offset** (e.g., GHG emissions) under defined market and accounting rules; offsetting tends to be treated as a residual mechanism after mitigation efforts, though debates persist about integrity. Local impacts (e.g., wastewater emissions) are generally regulated rather than offset. | **Rejects offsetting** as the default response for local biodiversity impacts; instead, local ecological degradation is treated as something that must be managed on-site through design and operations. Any use of biodiversity credits or offsets is positioned as secondary and subject to stronger safeguards, given known scientific concerns about equivalence and verification in biodiversity markets. |
| **Reporting** | **Public disclosure** is expected or required, relying on standardized metrics and frameworks (CSRD, GRI, IFRS/ISSB, TCFD, ESG ratings), enabling benchmarking and external assurance. | Reporting is primarily **internal monitoring** for control; external reporting is optional and, when used, can be aligned with TNFD for structure, but indicators and time measurements are defined by managers to reflect local context and feasibility rather than cross-company comparability. |
| **Results and accounta-bility** | **Accountability is compliance-based**: firms must take actions necessary to meet legal and standard requirements, typically expressed in measurable indicators that can be audited; failure can lead to sanctions. | **Accountability is based on action and management**: facilities can plausibly improve the habitat within their perimeter, but biodiversity outcomes depend on external ecological dynamics beyond management control. The expected outcome is credible implementation and adaptive improvement, not expressed as precise, universal metrics. |

## SM 3. Practical Guidelines

### 3.1 Action portfolios that “avoid net problems”

A facility-first N-P approach requires action portfolios that can deliver credible local improvement without relying on biodiversity “netting” or offsets as the default performance mechanism. This is achieved by prioritising interventions that act on pressures and habitat condition within the facility perimeter, where managerial control is strongest and evidence can be made auditable (Bull et al., 2020; Morrison-Saunders et al., 2024). The portfolio logic is explicitly non-compensatory: local biodiversity impacts should be addressed locally wherever feasible, because equivalence across habitats and contexts is contested and because distant compensation can substitute for, rather than complement, on-site stewardship (Bull et al., 2013; Simpson et al., 2022; Wauchope et al., 2024; zu Ermgassen et al., 2026).

In industrial settings, feasible interventions tend to cluster around a small number of controllable levers. Habitat-structure actions focus on improving vegetation composition and structure in managed areas to increase habitat quality and ecological function while respecting safety constraints. Disturbance-regime actions adjust mowing schedules, maintenance timing, and access routines to reduce chronic disturbance and allow habitat features to develop over time, recognising that industrial disturbances are recurrent and therefore governance must address routine patterns rather than one-off impacts (Morrison-Saunders et al., 2024). Lighting actions reduce night-time disturbance and disorientation pressures where feasible, especially in peripheral areas, by changing intensity, directionality, and operational schedules without affecting core safety requirements. Drainage and water-feature actions focus on runoff pathways and retention interfaces, where small design changes and maintenance practices can reduce habitat degradation and improve ecological function in perimeter zones. Connectivity actions emphasise micro-connectivity and permeability at the facility edge, improving habitat continuity and reducing barriers, while acknowledging that landscape-scale connectivity exceeds the facility’s control and therefore cannot be claimed as an outcome guarantee. Chemical-use actions reduce pressures from herbicides, pesticides, or surface treatments in managed areas where alternatives are feasible, again prioritising controllable pressure reduction over accounting.

Crucially, governance controls are treated as part of the action portfolio, not merely as enabling conditions. This includes contractor specifications, SOP integration, inspection routines, corrective actions, and budget continuity. Without these controls, ecological improvements are reversible, and reversibility is a central reason why facility-scale N-P must focus on persistence mechanisms (Boiral, 2007; Joy-Camacho and Thornhill, 2024). This governance-first framing also responds to the literature on corporate commitments and biodiversity offsetting, which highlights that implementation gaps and weak governance can undermine claimed outcomes even where headline ambition is high (de Silva et al., 2019; Bull and Strange, 2018; Rampling et al., 2023).

A final design requirement is proportional cost and operational compatibility, particularly for SMEs. Industrial N-P is intended to target perimeter and managed spaces without altering product characteristics or core production processes, enabling modest, staged investment and incremental scaling as learning improves (Boiral, 2007; Joy-Camacho and Thornhill, 2024). This proportionality is not only pragmatic; it is a credibility mechanism. If N-P is framed as requiring comprehensive biodiversity accounting, costly assurance, or large-scale offsets, SMEs may either avoid engagement or adopt low-evidence actions primarily for communication. A feasible portfolio lowers barriers to entry while maintaining rigour through documentation, persistence, and conservative claims (Boiral, 2016; Senanayake et al., 2024; Pardo et al., 2025).

### 3.2 Monitoring and claims discipline for managers

Monitoring and claims discipline are the point at which N-P either becomes credible or collapses into rhetoric. The framework therefore treats monitoring design as a governance decision, not a purely technical exercise. The first discipline is indicator parsimony: managers select a small set of repeatable indicators that can be observed consistently under industrial constraints and that reflect the intervention logic, rather than attempting to measure biodiversity comprehensively. This approach is consistent with evidence that indicator choice can materially affect interpretation and even societal support, and that metrics can conceal important differences between taxa and ecological functions if applied uncritically (Martínez-Jauregui et al., 2021; Marshall et al., 2024). It is also aligned with the critique that biodiversity cannot yet be reduced reliably to universal commensurable “units of nature”, and that premature precision can create false confidence and perverse incentives (Wauchope et al., 2024; Simpson et al., 2022; Nicholson et al., 2021).

A second discipline is repeatability through simple field protocols that suit SMEs. Photo-points are a particularly useful device because they create a time series of visual evidence that is easy to standardise, supports internal learning, and can be linked directly to intervention records without implying that biodiversity has been fully quantified. When photo-points are coupled with a basic register of actions and observations, the facility can demonstrate an evidence chain from intervention to habitat change to any observed ecological signals. This evidence chain matters because biodiversity reporting is susceptible to impression management, and auditors or stakeholders need traceable artefacts rather than narrative claims (Boiral, 2016; Blanco-Zaitegi et al., 2022).

A third discipline is explicit uncertainty statements. Managers should communicate that ecological signals are influenced by external drivers and time lags, that monitoring is designed for learning and directionality, and that observed changes do not imply guaranteed outcomes beyond the facility’s control (Morrison-Saunders et al., 2024). This does not weaken credibility; it strengthens it, because it prevents the category error of treating partial evidence as net outcome proof. The framework therefore distinguishes claims about actions from claims about outcomes. Action claims can be strong when evidence is strong: actions implemented, maintained, governed, and verified internally through routine controls. Outcome claims must remain bounded unless stronger verification exists.

This leads to the central claims rule: managers should not claim “net gain achieved”, “net positive impact delivered”, or equivalent outcome statements unless the organisation can defend baselines, equivalence assumptions, and permanence mechanisms at a standard consistent with the literature on offsets and nature markets. Where such standards are absent, net claims are vulnerable to challenge and can expose the firm to reputational and governance risks (Bull et al., 2013; Cares et al., 2025; zu Ermgassen et al., 2026). Instead, facility-scale N-P communication should prioritise disciplined, evidence-linked statements such as sustained habitat improvement actions, reduced pressures, and monitoring signals interpreted conservatively. This approach directly responds to the warning that N-P may become diluted into broad “positive impact” rhetoric unless claims are anchored in credible evidence and governance (Milner-Gulland, 2022; Stenseke, 2025; Buckley et al., 2025).

### 3.3 Interaction with disclosure and policy frameworks

A facility-scale N-P programme does not sit outside disclosure and policy frameworks; it supplies the operational content needed to make those frameworks meaningful in practice. However, there is a governance risk in the opposite direction: if N-P is absorbed into disclosure-only compliance, it can become a reporting exercise rather than a stewardship system. The framework therefore positions N-P as complementary to, but not reducible to, disclosure architectures such as Taskforce on Nature-related Financial Disclosures (TNFD) and EU sustainability reporting regimes.

TNFD provides a coherent structure for identifying and assessing nature-related dependencies, impacts, risks and opportunities, and it promotes the LEAP process (Locate, Evaluate, Assess, Prepare) as a practical sequence for analysis and disclosure (TNFD, 2023). Facility-scale N-P can populate each stage with operational content. Locate and Evaluate can be supported by facility baselines and perimeter mapping that identify the managed spaces and interfaces where pressures and opportunities occur. Assess can be linked to intervention logic that focuses on controllable pressures and habitat function, and Prepare can be managed through SOP changes, contractor controls, and monitoring routines embedded into EMS governance. In this way, TNFD can structure the “why” and “what” of nature-related management and disclosure, while N-P specifies the “how” of implementation in day-to-day operations (TNFD, 2023; Boiral, 2007).

Similarly, EU reporting requirements and standards increase expectations that firms disclose impacts, dependencies and actions on biodiversity and ecosystems. The framework’s contribution is that it creates an assurance-ready internal evidence chain. Rather than relying on general statements of intent, a facility can provide traceable records of actions, persistence mechanisms, and monitoring signals that support credible narratives under ESG and related reporting pressures (European Union, 2023; Blanco-Zaitegi et al., 2022). This approach addresses a core weakness in biodiversity reporting identified in the literature: the tendency for organisations to report what is difficult to verify and to optimise narratives under reputational incentives (Boiral, 2016). By anchoring disclosures in facility evidence, N-P helps shift biodiversity reporting from narrative substitution to operational transparency.

At the same time, the framework insists that disclosure is not a substitute for stewardship. Regulatory and reporting regimes can compel transparency, but they cannot by themselves ensure that maintenance routines, contractor practices, and disturbance regimes change in ways that improve local habitat condition. For this reason, the framework’s centre remains facility governance and operational embedding. Disclosure frameworks should be treated as external accountability mechanisms that reward transparency, while N-P provides the internal machinery that produces ecological directionality through controllable action. This division is particularly important for SMEs, which face administrative burdens and limited specialist capacity; a facility-appropriate method can prevent the false choice between “do nothing because it is complex” and “report ambition without operational content” (Joy-Camacho and Thornhill, 2024; Senanayake et al., 2024).

**3.4 A practical implementation model for SMEs**

Industrial Nature-Positive (N-P) for small and medium enterprises (SMEs) can be implemented as a minimum viable stewardship system organized around the Plan–Do–Check–Act (PDCA) cycle. This ordering fits the controllability principle: facilities are accountable for interventions and maintenance quality, while ecological outcomes are assessed through feasible proxies and directional signals rather than deterministic biodiversity claims.

**Plan (define scope, baseline, objectives, and the evidence chain)**

**1) Define system boundary and constraints**

- Map the facility perimeter and distinguish: (i) safety/no-go zones, (ii) operational interfaces (yards, verges, drainage), and (iii) candidate habitat zones.
- Record non-negotiable constraints (safety setbacks, firebreak requirements, access/visibility, security needs) that shape what is feasible and what must persist.

**2) Establish a pragmatic baseline (diagnostic, not accounting)**

- Describe current habitat types and condition using a simple typology (sealed surfaces, ruderal patches, managed lawn, shrub strips, swales, retention basins).
- Add a “disturbance profile” (mowing frequency, chemical use zones, lighting at night, noise peaks, traffic rhythms).
- Use screening tools where helpful for context, without converting baseline into rigid baseline-to-target accounting.

**3) Set objectives suited to uncertainty**

- Express objectives as site-based improvement intents (e.g., “increase habitat structure diversity in perimeter zones,” “reduce disturbance in habitat margins,” “increase continuity of flowering resources”), not as universal biodiversity units.
- Explicitly separate action accountability (what will be done and maintained) from outcome uncertainty (what biodiversity response may be observed).

**4) Design the evidence chain**

- Define two evidence layers: (i) intervention evidence (what is installed/changed and how maintained) and (ii) ecological signal evidence (proxy indicators and repeatable observations).
- Create the skeleton of an intervention register (action, location, intended function, maintenance needs, responsible role).

**Do (implement interventions and embed them into operations)**

**5) Implement a low-cost intervention portfolio**

- Habitat structure and vegetation: replace low-value lawn with meadow strips; add shrubs/herb layers; create microhabitats where safe.
- Connectivity and permeability: improve small-fauna permeability in fencing where compatible with security; connect patches via planted strips.
- Disturbance management: adjust mowing timing; reduce excessive night lighting and spill at vegetated margins; manage traffic rhythms near habitat zones where feasible.
- Water and drainage as habitat: enhance swales, retention basins, and pond edges for habitat value where safety permits.
- Nature-based additions around infrastructure: apply Nature-Based Solutions (NBS) in buffers and managed edges without disrupting production.

**6) Embed persistence mechanisms**

- Translate requirements into contractor specifications (mowing schedules, chemical restrictions, protected zones).
- Integrate N-P actions into existing maintenance planning and, where present, Environmental Management System routines (e.g., ISO 14001 and 20400 procedures) to reduce dependence on individual champions.

**Check (monitor proxies, verify persistence, interpret directional signals)**

**7) Run proxy monitoring on a feasible schedule**

- Habitat-condition proxies (quarterly/seasonal): vegetation cover/structure by zone; flowering continuity; invasive species flags; maintenance compliance.
- Disturbance proxies (monthly/seasonal): mowing frequency; chemical applications; lighting settings; disturbance events near habitat zones.
- Ecological signal proxies (seasonal): simple observations (pollinators, butterflies, birds) along repeatable walk routes; photographic repeat points for visual evidence.

**8) Apply an interpretation rule consistent with measurement limits**

- Use indicators to test whether the observed direction of change is consistent with the intervention logic and whether gains persist, without claiming that proxies equal a universal biodiversity metric.
- Record deviations, unexpected outcomes, and plausible external drivers (weather anomalies, landscape disturbances), maintaining transparency about partial controllability.

**9) Maintain credibility safeguards**

- Keep a short audit trail: intervention register updates + monitoring notes + photographs.
- Avoid absolute “net gain achieved” claims unless stronger measurement and verification are in place, consistent with known risks of impression management in biodiversity reporting.

**Act (learn, correct, scale, and optionally disclose)**

**10) Correct and adapt**

- Modify maintenance regimes, replace interventions that fail operationally, and address persistence issues (e.g., contractor drift, invasive species).
- Add one or two new interventions per cycle to scale gradually without increasing administrative burden.

**11) Institutionalize governance**

- Assign clear roles (site N-P champion within operations; periodic ecology support).
- Where feasible, include N-P in internal audits and corrective actions to stabilize continuity under staff turnover.

**12) Decide on proportional transparency**

- External reporting remains optional for SMEs; when chosen, use the evidence chain (interventions + proxy signals) to populate structured disclosures (e.g., TNFD-aligned narratives) without overstating comparability or certainty.
- Where feasible, consider a light external review (periodic site walk) to strengthen credibility without converting the program into a high-cost compliance system.

## SM 4. Menu of actions

This library supports the Do phase of the Plan–Do–Check–Act (PDCA) cycle for small and medium enterprises (SMEs) implementing Nature-Positive (N-P) practice at the facility scale. Actions are written to be operationally compatible and to fit Health, Safety and Environment (HSE) constraints. Expected ecological functions are expressed as plausible mechanisms (habitat condition, resources, connectivity, disturbance reduction) rather than guaranteed biodiversity outcomes.

**How to use this library**

Choose 3 to 6 actions for year 1 (low disruption), then add 1 to 2 actions per year.

**A. Vegetation and habitat structure**

| **Action type** | **Where applicable** | **Constraints / safety notes** | **Maintenance requirement** | **Expected ecological function** |
| --- | --- | --- | --- | --- |
| **Convert low-value lawn to meadow strip (native/locally appropriate mix)** | Road verges, perimeter greens, buffer strips, low-footfall areas | Maintain sightlines near junctions /security; avoid pollen-allergen sensitive areas if relevant; fire risk review in dry climates | 1–2 cuts/year; remove arising if nutrient reduction desired; reseed patches; invasive plant checks | Increased floral resources; higher vegetation structural diversity; improved invertebrate habitat |
| **Create “refugia zones” (no-mow micro-areas)** | Corners, behind buildings, fence margins, embankments | Avoid pest harborage near food areas; ensure access for inspection; don’t block drainage | Mark on site map; mow edge “frame” for neatness; seasonal litter removal | Safe shelter and overwintering habitat; microclimate diversity |
| **Plant native shrub belt / hedgerow (mixed structure)** | Perimeter buffers, along fences, between hard standing and boundary | Keep clearance from utilities; ensure security patrol access; avoid species that attract large nuisance fauna near operations | Annual formative pruning; replace failures; weed control for first 2 seasons | Nesting/shelter; connectivity corridor; windbreak microhabitats |
| **Add layered planting (shrub + herb + grass)** | Where there is enough width (≥2–3 m) | Don’t obstruct emergency routes; maintain firebreak distances if required | Seasonal thinning; invasive control; mulch top-up | Higher habitat complexity; longer resource season |
| **Install pollinator patches (high-diversity flowering beds)** | Entrances, office areas, low-risk showcase zones | Avoid near sensitive intake vents; confirm no conflict with allergen policies | Seasonal weeding; replanting; irrigation in establishment year | Nectar/pollen continuity; supports pollinators and natural enemies |
| **Introduce native climbers on suitable structures** | Fences/walls away from equipment | Avoid growth into electrical boxes/ventilation; manage structural loading | Annual cut-back; ensure no encroachment | Vertical habitat, shade, insect resources |
| **Create deadwood habitat (logs/branches)** | Quiet corners, shrub belts, designated habitat zone | Fire risk assessment; avoid termite risk where relevant; keep away from building fabric if pests are a concern | Replace as it decomposes; keep tidy perimeter | Saprophytic habitat; beetles/fungi; refuge for small fauna |
| **Create rock/stone refuges (“hibernacula”)** | Dry margins, embankments, near shrub belts | Must not create trip hazards; keep away from vehicle routes | Minimal; check stability; remove litter | Shelter/thermal refuges; overwintering sites |
| **Install sand/soil bare patches (for ground-nesting bees)** | Sunny, well-drained margins | Erosion control; avoid dust nuisance; keep away from operations sensitive to particulate | Keep patch open (remove vegetation); replenish annually if needed | Nesting substrate; supports pollinators |
| **Add “green edges” to hard standing (planted strips)** | Car parks, paved boundaries, perimeter of yards | Maintain vehicle clearance; avoid hiding spots near security-sensitive zones | Weed control; replanting; irrigation early | Increases edge habitat; reduces fragmentation |

**B. Connectivity and permeability**

| **Action type** | **Where applicable** | **Constraints / safety notes** | **Maintenance requirement** | **Expected ecological function** |
| --- | --- | --- | --- | --- |
| **Wildlife-permeable fencing sections (small-fauna gaps /underpasses)** | Long perimeter fences where security allows | Security risk assessment; exclude high-security sectors; ensure gaps do not allow unauthorized access | Quarterly inspection; clear blockages | Improves micro-connectivity for hedgehog/small mammals/amphibians |
| **“Green corridor” strip connecting habitat patches** | Within site: between buffers, ponds, shrub belts | Maintain access for emergency vehicles; keep clear of hazardous zones | Same as meadow/shrub maintenance | Reduces isolation; supports movement and colonization |
| **Vegetated stepping stones (small patches at intervals)** | Where continuous corridors not feasible | Avoid nuisance near loading bays; maintain visibility | Seasonal maintenance | Supports dispersal; provides rest/refuge nodes |
| **Canopy continuity (tree/shrub alignment)** | Along boundaries or internal roads | Avoid overhead conflicts with power lines; root conflicts with underground utilities | Pruning; root barrier where needed | Increases structural connectivity and microclimate stability |

**C. Water, drainage, and wet habitat features**

| **Action type** | **Where applicable** | **Constraints / safety notes** | **Maintenance requirement** | **Expected ecological function** |
| --- | --- | --- | --- | --- |
| **Nature-friendly swale design (vegetated drainage channels)** | Stormwater management zones | Must meet drainage capacity and flood requirements; mosquito risk management plan | Seasonal vegetation management; sediment removal | Wet–dry habitat mosaic; supports insects and plants; improves water quality |
| **Retention basin edge enhancement (gentle slopes + planting)** | Existing basins/ponds | Drowning risk controls; fencing/signage; comply with water safety standards | Annual edge maintenance; invasive aquatic control | Amphibian/insect habitat; waterbird foraging (context-dependent) |
| **Rain gardens / bioretention cells** | Car parks, roof runoff points | Avoid interference with foundations; ensure overflow routes | Seasonal mulching/plant replacement; sediment clearing | Pollutant filtration; moisture habitat; floral resources |
| **“No-mow” buffer around wet features** | Around ponds/swales | Maintain inspection access; avoid creating hidden hazards | Defined mowing boundary; litter control | Reduces disturbance; improves riparian vegetation structure |
| **Floating vegetation islands (where appropriate)** | Larger basins | Must not obstruct outlets or maintenance access | Periodic anchor checks; plant replacement | Refuge habitat; water quality support; invertebrate colonization |

**D. Disturbance regime management**

| **Action type** | **Where applicable** | **Constraints / safety notes** | **Maintenance requirement** | **Expected ecological function** |
| --- | --- | --- | --- | --- |
| **Mowing schedule change (reduce frequency; avoid flowering peak)** | Meadows, margins, buffers | Fire risk review; tick/pest considerations; maintain access/sightlines where needed | Implement schedule; record deviations | Increases flowering and seed set; reduces disturbance; improves invertebrate habitat |
| **“Quiet zones” (reduced noise/disturbance periods)** | Adjacent to key habitat patches | Must not compromise production or safety; set realistic windows | Procedure inclusion; signage | Reduces chronic disturbance; supports breeding/foraging |
| **Traffic routing away from habitat zones** | Internal roads near green strips | Must preserve emergency routes; align with logistics | Marking/signage; periodic compliance checks | Reduces disturbance and mortality risk |
| **Seasonal timing rules for major grounds works** | Any landscaping/excavation | Must not conflict with safety/operations; consider nesting season | Planning control; contractor clauses | Avoids destroying nests/larval stages; reduces one-off impacts |

**E. Lighting and night-time management**

| **Action type** | **Where applicable** | **Constraints / safety notes** | **Maintenance requirement** | **Expected ecological function** |
| --- | --- | --- | --- | --- |
| **Reduce spill lighting (shielding, directional fixtures)** | Perimeter lights near vegetation | Must meet security and safety illumination levels | Periodic alignment checks | Reduces insect disorientation and predation risk; supports nocturnal fauna |
| **Curfew or dimming schedule in low-risk zones** | Office car parks, secondary yards | Security sign-off required | Timer settings review; incident exception logging | Lower night disturbance; supports bats/insects |
| **Warm-spectrum lighting in habitat-adjacent zones (where permitted)** | Near green edges | Ensure compatibility with standards; procurement constraints | As per fixture life cycle | Reduces ecological impact relative to high-blue content lighting (context-dependent) |

**F. Chemical use and pollution prevention**

| **Action type** | **Where applicable** | **Constraints / safety notes** | **Maintenance requirement** | **Expected ecological function** |
| --- | --- | --- | --- | --- |
| **Reduce herbicide in habitat zones (mechanical/targeted alternatives)** | Meadows, shrub belts, swales | Must comply with safety/invasive control needs; worker protection | Revised procedures; spot-treatment records | Protects non-target plants/invertebrates; improves habitat integrity |
| **Integrated Pest Management (IPM) for grounds** | Landscaped areas | Must not compromise biosecurity rules for certain industries | Monitoring + threshold-based actions | Reduces broad-spectrum pesticide pressure |
| **Dust suppression near habitat zones (operational)** | Unsealed surfaces | Balance with water use; avoid chemical suppressants harmful to biota | Routine during dry periods | Protects vegetation and invertebrates; improves plant establishment |
| **Spill and runoff containment upgrades** | High-risk operational zones | Must meet regulatory requirements; engineering sign-off | Inspection and maintenance schedule | Reduces toxic exposure; protects wet features |

**G. Soil and substrate management**

| **Action type** | **Where applicable** | **Constraints / safety notes** | **Maintenance requirement** | **Expected ecological function** |
| --- | --- | --- | --- | --- |
| **Topsoil preservation during works** | Construction/retrofit projects | Must not obstruct construction safety | Soil storage plan; re-spread protocols | Retains seed bank and soil biota; improves restoration outcomes |
| **Reduce compaction in habitat zones (designated no-vehicle)** | Meadows, buffers | Must maintain emergency access | Physical barriers/signage; periodic enforcement | Improves infiltration and plant establishment |
| **Add micro-topography (small mounds/depressions)** | Habitat zones | Must not create trip hazards; erosion control | Occasional reshaping | Creates microclimates and niches |

**H. Invasive species and ecological hygiene**

| **Action type** | **Where applicable** | **Constraints / safety notes** | **Maintenance requirement** | **Expected ecological function** |
| --- | --- | --- | --- | --- |
| **Invasive plant surveillance and rapid response** | All vegetated zones | Follow local regulations; protect workers from allergens | Seasonal survey; removal log | Prevents biodiversity loss via dominance; improves habitat condition |
| **Clean-down protocols for contractors (soil/seed transfer)** | Works moving soil/plant | Must be practical; integrate into contract | Procedure inclusion; checks | Reduces introduction/spread of invasives and pathogens |

**I. Built micro-habitats and biodiversity features**

| **Action type** | **Where applicable** | **Constraints / safety notes** | **Maintenance requirement** | **Expected ecological function** |
| --- | --- | --- | --- | --- |
| **Nest boxes (birds)** | Quiet edges, shrub belts | Avoid near sensitive operations; nuisance management | Annual cleaning/check | Supports cavity nesters (context-dependent) |
| **Bat boxes (where relevant)** | Tree lines/buildings (approved zones) | Legal protections often apply; specialist input recommended | Periodic inspection (specialist) | Roosting support; strengthens nocturnal trophic links |
| **Insect hotels (small-scale demonstration)** | Low-risk landscaped zones | Limited ecological value if poorly designed; avoid public hazard | Annual upkeep | Public engagement; small refuge value |
| **Green roofs/walls (Nature-Based Solutions)** | Suitable roofs/walls | Structural loading; fire safety; waterproofing; maintenance access | Seasonal maintenance; irrigation early | Adds habitat area; cooling and stormwater benefits (context-dependent) |

**J. Governance and persistence mechanisms (often the difference between “one-off” and “real”)**

| **Action type** | **Where applicable** | **Constraints / safety notes** | **Maintenance requirement** | **Expected ecological function** |
| --- | --- | --- | --- | --- |
| **Contractor biodiversity clauses** | Grounds maintenance contracts | Must be enforceable and measurable | Annual contract review | Preserves gains by controlling maintenance behavior |
| **“Habitat protection map” in work permit system** | Sites with permit-to-work | Must be simple and visible | Update after changes | Avoids accidental damage; institutionalizes stewardship |
| **Training toolbox talks (seasonal)** | Grounds/contractor teams | Keep short; focused | 2–4 per year | Reduces inadvertent impacts; improves compliance |

## SM 5. Monitoring templates

This section provides three practical templates to support facility-scale N-P implementation under the Plan–Do–Check–Act (PDCA) cycle. Templates are designed for small and medium enterprises (SMEs): low burden, auditable, and compatible with operational constraints.

1. **Intervention register template**

Table 1: Intervention register

| **Field** | **What to record** | **Example** |
| --- | --- | --- |
| **Intervention ID** | Unique code | NP-INT-001 |
| **Intervention name** | Short action title | Meadow strip conversion |
| **Action category** | Choose one | Vegetation / Water / Lighting / Connectivity / Disturbance / Soil / Governance |
| **Location / zone** | Site zone name + map ref | Perimeter East, Zone E3 |
| **GPS (optional)** | If available | 41.40338, 2.17403 |
| **Installed / start date** | When implemented | 15/03/2026 |
| **Responsible owner** | Role (not person) | Facilities manager |
| **Contractor involved** | Yes/no + contractor name | Yes — Green Works Ltd |
| **Intended ecological function** | Mechanism, not outcome | Increase floral resources and habitat structure |
| **Constraints / safety notes** | HSE constraints | Maintain sightlines; firebreak 1 m |
| **Maintenance standard** | What “good” looks like | 2 cuts/year; remove arisings; no herbicide |
| **Maintenance cadence** | Frequency | April + September |
| **Materials/specification** | Mix/seed/spec | Native mix A; 20 species |
| **Monitoring proxy (primary)** | One simple proxy | Flowering continuity score |
| **Monitoring proxy (secondary)** | Optional | Vegetation height band |
| **Photo-point ID** | If used | PP-E3-01 |
| **Status** | Planned / Active / Modified / Removed | Active |
| **Change log note** | If modified | Reduced mowing near gate due to access |

2. **Monitoring sheet template**

Use this sheet during “Check” walks. One sheet can cover one zone, one intervention, or a whole route. Keep methods consistent (same route, similar timing each year).

**2a. Habitat condition and intervention persistence (seasonal)**

Table 2a: Habitat condition monitoring

| **Field** | **What to record** | **Response options / notes** |
| --- | --- | --- |
| **Date / time** | When observed | YYYY-MM-DD; HH:MM |
| **Observer** | Role or initials | FM / HSE / contractor |
| **Weather note** | Brief | Dry / wet / windy |
| **Zone / intervention IDs** | Link to register | Zone E3; NP-INT-001 |
| **Intervention present?** | Has it persisted? | Yes / Partially / No |
| **Maintenance completed as planned?** | Check cadence | Yes / No / Partial |
| **Vegetation structure (simple)** | Choose band | Short (<10 cm) / Medium (10–30) / Tall (>30) / Mixed |
| **Flowering continuity (seasonal)** | Quick score | 0 none / 1 low / 2 moderate / 3 high |
| **Bare ground patches** | Presence | None / few / many |
| **Invasive species flags** | Any priority invasives | None / suspected / confirmed (name) |
| **Litter / dumping** | Note | None / minor / major |
| **Evidence of disturbance** | e.g., trampling, mowing drift | None / minor / major |
| **Photo taken?** | Photo-point match | Yes (PP-E3-01) / No |
| **Notes / deviations** | Free text | Contractor cut early near gate |

**2b. Disturbance and operational controls (monthly or quarterly)**

Table 2b: Disturbance controls monitoring

| **Field** | **What to record** | **Response options / notes** |
| --- | --- | --- |
| **Date** |  | YYYY-MM-DD |
| **Zone(s)** |  | E3; perimeter strip |
| **Mowing frequency since last check** | Count | # events |
| **Chemical applications since last check** | Type/date | None / spot / broad (details) |
| **Night lighting setting (if relevant)** | On/off/dim | On / Dim / Curfew |
| **Access compliance** | Vehicles in habitat zone? | Yes / No (describe) |
| **Contractor compliance issues** | Any breaches | None / minor / major |
| **Corrective action needed?** | Trigger | Yes / No |

**2c. Ecological signal observations (optional, seasonal)**

Table 2c: Ecological signals (proxy observations)

| **Field** | **What to record** | **Response options / notes** |
| --- | --- | --- |
| **Date / route ID** | Repeatable route | Route R1 |
| **Duration** | Minutes | 15 |
| **Pollinator activity (quick index)** | Visual abundance | 0 none / 1 low / 2 moderate / 3 high |
| **Butterfly sightings** | Count (rough) | # |
| **Bird activity** | Presence | None / some / high |
| **Notable species (if known)** | Optional | e.g., “swallow” |
| **Evidence of nesting/breeding** | If observed | Yes / No |
| **Notes** | Free text | High activity on flowering patch |

3. **Annual PDCA review form (management summary)**

**3a. Annual PDCA review cover sheet**

Form 3a. Annual review summary

Site:

Year / period covered:

Review date:

Review lead (role):

Participants (roles): Operations / HSE / Facilities / Contractor

**3b. PLAN review (what we intended)**

Boundary/constraints changes this year:

None / Yes (describe)

Objectives set last review (max 3):
1)
2)
3)

New risks or dependencies identified (if any):

**3c. DO review (what we implemented)**

Table 3c: Intervention implementation review

| Metric | Value |
| --- | --- |
| Interventions planned | # |
| Interventions implemented | # |
| Interventions modified | # |
| Interventions removed | # |
| Contractor clauses updated? | Yes / No |

**3d. CHECK review (what we observed)**

Table 3d: Monitoring completeness and signals

| **Item** | **Status** |
| --- | --- |
| **Habitat-condition monitoring done as planned** | Yes / Partial / No |
| **Disturbance monitoring done as planned** | Yes / Partial / No |
| **Photo-points repeated** | Yes / Partial / No |
| **Ecological signals recorded (optional)** | Yes / No |

Directional assessment (choose one per objective):

- Objective 1: Improving / Stable / Worsening / Unclear
- Objective 2: Improving / Stable / Worsening / Unclear
- Objective 3: Improving / Stable / Worsening / Unclear

Top 3 issues identified (if any):
1)
2)
3)

**3e — ACT review (what we will change next cycle)**

Corrective actions (fix persistence/compliance) — max 5:

Action / owner / deadline

Adaptations (change intervention design) — max 5:

Next-cycle additions (1–2 new interventions recommended):

Proposed intervention:

Expected function:

Constraints:

Maintenance plan:

Decision on transparency (optional):

External reporting: None / limited / TNFD-aligned narrative / other

External review: None / site walk / third-party check

**3f . Claims discipline (tick-box)**

Before any communication, confirm:

We can show an up-to-date intervention register

We can show maintenance evidence (work orders/contractor logs)

We have proxy monitoring records and/or photo-point evidence

We avoid “net gain achieved” claims without stronger verification

We describe uncertainty and external drivers where relevant

## SM 6. Examples

This is a chemical company near Madrid where six potential areas have been identified, pending detailed analysis. Areas 1, 2, and 3 are suitable for planting vegetation and trees, with a total surface area of 2,000 m². Areas 4 and 6 are suitable for green walls of up to 1,600 m², and area 5 could accommodate a 500 m² green roof.

Figure 5: Company A. Current building

Figure 6: Company A. Suggested changes

Additional actions

- Refuge zones
- Insect hotels
- Pollinator patches
- Deadwood habitats
- Rock stone refuges

This is a company that manufactures products for hospitals, where in points 1, 2 and 3 plants, trees, and animal shelters can be placed in 1,100 m^2^, and in points 4 and 5 green walls of 1,000 m^2^.

Figure 7: Company B. Current building

Figure 8: Company B. Suggested changes

Additional actions

Attenuating the night light

Replace light by thermal and movement sensors

Nest boxes

1. Universidad Europea de Madrid (UEM) Department of Veterinary Medicine. Faculty of Biomedical and Health Sciences. Villaviciosa de Odón. Madrid (Spain) [*angela.martin@universidadeuropea.es*](mailto:angela.martin@universidadeuropea.es) ORCID 0009-0005-5719-5405
2. ^(c)^ Universidad Politécnica de Cartagena (UPCT) Member of European University of Technology EUT+, Business Economy Department. Campus Muralla del Mar. Cartagena (Spain) [*Francisco.Campuzano@upct.es*](mailto:Francisco.Campuzano@upct.es) ORCID 0000-0003-1141-5810
3. ESADE Business School. Barcelona (Spain). [*david.lopez1@esade.edu*](mailto:david.lopez1@esade.edu) ORCID 0000-0003-4459-275X
